# The Autophagy–Inflammasome Axis as a Molecular Switch: From Persistent Inflammation to Vascular Remodeling in IVIG-Resistant Kawasaki Disease

**DOI:** 10.3390/ijms27146405

**Published:** 2026-07-18

**Authors:** Rong Zhang, Jiaqi Zhang, Yanzhi Yang, Ya Wang, Haijun Cao

**Affiliations:** Institute of Blood Transfusion, Chinese Academy of Medical Sciences & Peking Union Medical College, Chengdu 610052, China; s2024019015@student.pumc.edu.cn (J.Z.); s2025019011@student.pumc.edu.cn (Y.Y.); wymelinda@ibt.pumc.edu.cn (Y.W.); haijuncao@ibt.pumc.edu.cn (H.C.)

**Keywords:** IVIG-resistant Kawasaki disease, coronary artery lesions, autophagy, endothelial-to-mesenchymal transition, vascular remodeling

## Abstract

Intravenous immunoglobulin (IVIG) resistance occurs in 10–20% of children with Kawasaki disease (KD) and is associated with a 3- to 5-fold higher risk of coronary artery lesions (CALs). Yet the mechanistic basis for why some patients progress from reversible inflammation to irreversible vascular damage after IVIG remains poorly understood. Most existing reviews have focused on risk prediction rather than the mechanistic chain linking resistance to CALs. Here, we synthesize current evidence across three interconnected pathways. First, autophagy dysfunction—particularly impaired mitophagy—sustains inflammation through cGAS-STING activation. Second, neutrophil extracellular traps (NETs) play a controversial role in KD vasculitis, with PAD2 and PAD4 possibly acting redundantly via the NLRP3 inflammasome. Third, endothelial-to-mesenchymal transition (EndMT), driven by the IL-1β/TNF axis and the USP7-TGFβ2/SMAD pathway, emerges as a core event in vascular remodeling. Building on these findings, we propose the “autophagy–inflammasome axis” as a candidate molecular switch that dictates whether inflammation resolves or persists. This hypothesis is actionable: it generates three explicit, testable predictions linking autophagic integrity to inflammatory outcomes and therapeutic response. Direct experimental validation in IVIG-resistant KD models and patient samples is now urgently needed. This review provides a systematic framework for understanding how IVIG resistance transitions to irreversible CALs. It also identifies candidate biomarkers (e.g., S100A12, mtDNA, and MCM8) and therapeutic targets (autophagy inducers, NLRP3 inhibitors, USP7 inhibitors, and anakinra) that could enable earlier intervention.

## 1. Introduction

Kawasaki disease (KD) is an acute, self-limited vasculitis of unknown etiology that predominantly affects children under five years of age, with coronary artery lesions (CALs) as its most serious complication [1,2,3]. The global incidence of KD varies considerably across populations, with the highest rates reported in East Asian countries (e.g., Japan: approximately 300–400 per 100,000 children under five years) and increasing incidence in Western countries [1,2]. Since Furusho et al. [4] first demonstrated that high-dose intravenous immunoglobulin (IVIG) significantly reduces the incidence of coronary artery aneurysms, IVIG combined with aspirin has become the standard of care [2,5,6]. Nevertheless, approximately 10–20% of patients do not respond to IVIG (i.e., IVIG resistance) [7,8,9], and their risk of CALs is 3- to 5-fold higher than that of responders [2].

The mechanisms underlying the beneficial effects of IVIG in KD are multifactorial and not fully elucidated. Proposed mechanisms include: (1) Fcγ receptor (FcγR)-mediated modulation of immune complex clearance and inhibition of activating FcγR signaling [10,11]; (2) neutralization of pathogenic antibodies and suppression of pro-inflammatory cytokine production [12]; (3) induction of anti-inflammatory effects via sialylated IgG and DC-SIGN engagement [13]; and (4) modulation of T-cell and monocyte/macrophage functions [14]. The failure of IVIG in 10–20% of patients suggests that these regulatory mechanisms are insufficiently engaged in resistant individuals, possibly due to genetic variation in FcγR genes, altered IgG glycosylation patterns, or dysregulation of the autophagy–inflammasome axis that sustains inflammation despite exogenous immune modulation.

“Mechanistic transition” here means the molecular and cellular processes by which acute reversible inflammation shifts to chronic structural vascular remodeling in IVIG-resistant KD. Existing reviews have largely focused on risk factors or predictive models for IVIG resistance [8,15,16,17], but the mechanistic pathway from resistance to CALs remains poorly integrated. Although autophagy dysfunction, neutrophil extracellular trap (NET) formation, and endothelial-to-mesenchymal transition (EndMT) have been reported in KD [18,19,20,21,22], their bridging roles between IVIG resistance and CALs remain unsystematically reviewed. A recent authoritative annual review by Noval Rivas and Arditi comprehensively updated the pathophysiology and emerging therapeutic approaches in KD but did not specifically address the mechanistic transition from reversible inflammation to irreversible vascular remodeling in IVIG-resistant patients [23]. This review focuses on filling that gap.

IVIG resistance is not merely a quantitative extension of standard KD but appears to represent a distinct biological phenotype characterized by: (1) more intense and sustained activation of the NLRP3 inflammasome and IL-1β production [24,25]; (2) a higher proportion of neutrophils and monocytic myeloid-derived suppressor cells (MDSCs) with T-cell exhaustion [23]; (3) genetic predispositions that may affect immune regulation (e.g., ITPKC, CASP3, and FCGR2A variants) [26,27,28]; and (4) more pronounced and prolonged endothelial activation that predisposes to irreversible vascular remodeling [21,29]. This subgroup thus offers a unique window into failed inflammation resolution and subsequent vascular remodeling—processes largely bypassed in IVIG-responsive patients.

This review addresses three questions:

- Why is the inflammatory network persistently activated in the IVIG-resistant state?
- How do autophagy, NETs, and EndMT connect inflammation to vascular remodeling?
- Is there a molecular switch that determines the transition from “reversible inflammation” to “irreversible injury”?

Throughout this review, the “autophagy–inflammasome axis” refers to the functional balance between autophagic activity (especially mitophagy) and NLRP3 inflammasome activation. When autophagy is intact, it clears damaged mitochondria and degrades NLRP3 components, suppressing IL-1β production and promoting resolution. When autophagy is defective, accumulated mtDNA activates the cGAS-STING pathway, driving sustained NLRP3 activation and IL-1β release. We hypothesize that this axis may act as a binary switch—“off” (resolution) or “on” (persistent inflammation → remodeling) —though direct evidence in IVIG-resistant KD is currently lacking and requires prospective validation.

This narrative review synthesizes evidence on the autophagy–inflammasome axis in IVIG-resistant Kawasaki disease (KD) vasculopathy. We searched PubMed/MEDLINE, Web of Science, and Scopus for articles published from January 1980 to March 2026, using combinations of keywords including “Kawasaki disease”, “IVIG resistance”, “autophagy/mitophagy”, “NLRP3 inflammasome/cGAS-STING”, “neutrophil extracellular traps”, “EndMT”, “vascular remodeling”, and “coronary artery lesions”. We included peer-reviewed original research, clinical studies, case series, and relevant reviews. Prioritization was given to studies directly addressing mechanistic links between autophagy/mitophagy and inflammation in KD, IVIG resistance, coronary outcomes, or therapeutic targeting of this axis. Evidence from broader inflammatory contexts was included only when KD-specific data were lacking, and such evidence is explicitly identified as extrapolated in the text. Conference abstracts, unpublished data, and non-English articles were excluded. Reference lists of included articles were manually screened for additional relevant studies.

## 2. Persistent Inflammation in IVIG-Resistant KD

### 2.1. T-Cell Subset Imbalance and Cytokine Storm

Acute KD features significant T-cell immune abnormalities. Children with CALs have elevated peripheral blood CD14^+^ monocyte counts [30]. Single-cell transcriptomics reveals deeper immune heterogeneity. Feng et al. (2025) reported that IVIG non-responders showed a significantly increased neutrophil proportion, a markedly decreased CD8^+^ T cell population, and T-cell exhaustion characterized by substantially elevated PD-1^+^ cells [31]. Furthermore, Feng et al. observed expansion of monocytic MDSCs in IVIG non-responders, which suppressed T-cell function and promoted inflammation. Using machine learning, Wang et al. (2025) identified six inflammation-related diagnostic biomarkers with a combined model AUC of 0.96 [32]. These findings suggest immune cell subset differences may be associated with IVIG resistance.

A recent multiomics review by Ahn and Kang synthesized high-throughput profiling data in KD and highlighted several convergent mechanistic axes that are highly relevant to IVIG resistance, including IL-1/IL-6-neutrophil programs, Fcγ receptor signaling related to IVIG pharmacodynamics, Ca^2+^/NFAT-dependent T-cell activation, and endothelial–extracellular matrix remodeling associated with coronary outcomes [33]. The IL-1/IL-6-neutrophil axis directly links to the NLRP3 inflammasome-derived IL-1β discussed above, and Fcγ receptor signaling may influence IVIG responsiveness through genetic variants such as FCGR2A (Table 1). These findings from multiomics studies support the three mechanisms discussed below.

### 2.2. Genetic Susceptibility and Immune Dysregulation

Genetic factors are associated with KD susceptibility and IVIG resistance. Genome-wide association studies have identified multiple loci that mediate disease susceptibility and CALs through immune dysregulation [41,42]. These are summarized by the pathways presented in Table 1.Beyond these established loci, emerging evidence implicates Fcγ receptor dysfunction and complement activation in IVIG resistance. FcγRs play a critical role in IVIG’s anti-inflammatory mechanism, as IVIG is thought to exert its effects partly through competitive binding to FcγRs, thereby reducing the binding of pathological immune complexes [10,43]. Genetic variations at the FCGR2/3 locus have been associated with KD susceptibility; however, a large multi-cohort study by Uittenbogaard et al. (2024) involving 1167 KD cases found that FCGR2/3 polymorphisms were not significantly associated with IVIG resistance or CALs risk in meta-analyses, apart from a possible association in a Polish cohort for the FCGR3B-NA2 haplotype [10]. By contrast, studies have reported that elevated FcγRIIA expression and FcγRIIA/IIB mRNA expression ratios are associated with IVIG resistance and CALs formation [44], and hypomethylation of FcγR2B has been linked to IVIG resistance [45]. These discrepant findings suggest that FcγR-mediated mechanisms may be context-dependent and influenced by epigenetic regulation or disease stage.

Complement activation has been implicated in KD pathophysiology, as demonstrated by Atici et al. in a murine model [46]. However, direct evidence linking complement activation to IVIG resistance in clinical settings remains limited. While complement levels have been reported to differ between IVIG-sensitive and non-responsive groups [47], the mechanistic role of complement in driving IVIG resistance and vascular injury requires further investigation. The present review focuses on the autophagy–inflammasome axis, NETs, and EndMT as three interconnected pathways that we consider most directly supported by current mechanistic evidence in IVIG-resistant KD.

Among these loci, ITPKC, CASP3, FCGR2A, and TSPAN5 have been directly linked to IVIG resistance, whereas MCM8 and other mitochondria-related genes are associated with KD susceptibility, but their relationship with resistance remains unclear. Significant heterogeneity exists, and genetic risk scores based on these loci show good predictive value for severe CALs.

### 2.3. Inflammasome Activation and Pyroptosis 

NLRP3 inflammasome activation is a central event in KD vasculitis and is persistently activated in the IVIG-resistant state. Maury et al. (1988) first reported that serum IL-1β levels were elevated 4- to 6-fold in acute KD and even higher in those with coronary artery aneurysms [48]; Noval Rivas et al. highlighted the core pathogenic role of IL-1β in KD coronary arteritis [24]. Differences in absolute IL-1β values across studies may reflect variations in detection methods, disease stage, or sample processing.

Pyroptosis is a key mechanism for IL-1β maturation and release. Pyroptosis-related molecules are activated during the acute phase of KD, but the degree and duration of activation are greater in IVIG-resistant patients. In a prospective study of 144 children with KD, Li et al. (2024) found that CASP1 mRNA expression was significantly lower in the IVIG-resistant group, and a CASP1-based model achieved 91.7% sensitivity for predicting resistance [49]. Wang et al. (2025) reported that serum levels of pyroptosis-associated proteins (ASC, caspase-1, IL-1β, IL-18, and GSDMD) were significantly elevated in KD patients, with IL-1β positively correlated with CRP [50]. Mechanistically, Jia et al. demonstrated using co-culture systems and a KD mouse model that the HMGB1/RAGE/cathepsin B pathway activates the NLRP3 inflammasome and induces endothelial cell pyroptosis [51]. Thus, targeting NLRP3 inflammasome-mediated pyroptosis represents a potential therapeutic strategy for IVIG-resistant patients.

### 2.4. The S100A12-TLR4-MYD88 Axis

The S100A12-TLR4-MYD88 axis is central to the KD inflammatory network. Feng et al. found a stronger inflammatory response driven by this axis in IVIG non-responders; mechanistically, S100A12 activates the TLR4-MYD88-NF-κB axis, driving a sustained inflammatory response that may override IVIG’s anti-inflammatory effects [31]. Wu et al. (2024) reported that mRNA expression of S100A12 and MYD88 in PBMCs were significantly elevated in the IVIG-resistant group; a relative S100A12 expression ≥10.224 was an independent risk factor for IVIG resistance [52]. A 2025 study identified MYD88 and S100A12 as key pyroptosis-related genes in KD and suggested, based on molecular docking, that Atogepant, Ubrogepant, and Zanubrutinib might target these molecules [50]; however, these computational predictions require experimental validation.

The persistent inflammation described above does not resolve spontaneously in IVIG-resistant patients. Instead, it triggers downstream mechanisms that drive the transition to vascular remodeling.

## 3. Mechanistic Pathways from Inflammation to Vascular Remodeling

### 3.1. Autophagy Dysfunction and Impaired Mitophagy

Impaired mitophagy—a key form of autophagy dysfunction—drives the transition from acute reversible inflammation to chronic irreversible vascular remodeling early in the disease course.

#### 3.1.1. Bidirectional Regulation of Autophagy and Evidence in KD

Autophagy exerts bidirectional regulation on vascular inflammation. Physiologically, autophagy clears damaged organelles and suppresses inflammation [53,54]; pathologically, abnormal autophagic flux promotes persistent inflammation and tissue injury.

Studies in KD have reported divergent directions of autophagic change. Qin et al. (2021) detected significantly increased autophagic flux (elevated LC3-II/I ratio) in PBMCs from KD children; these PBMCs induced autophagy in human coronary artery endothelial cells (HCAECs) and promoted chemokine and pro-inflammatory cytokine secretion, an effect partially reversed by the autophagy inhibitor 3-methyladenine (3-MA) [55]. In contrast, Marek-Iannucci et al. (2021) observed impaired autophagy in the myocardial tissue of LCWE-induced KD model mice, characterized by a significantly increased LC3-II/I ratio and markedly increased p62 accumulation; metformin treatment substantially reduced coronary arteritis scores [19]. These differences suggest that the role of autophagy in KD is cell-type specific and temporally dynamic. A recent study by Singh et al. (2026) further demonstrated that IVIG remodels innate immune cell communication and induces differential autophagy pathways in KD, highlighting the complexity of autophagy regulation in response to therapy [18].

#### 3.1.2. Mitophagy and cGAS-STING Pathway Activation

Genetic variants in mitophagy-related genes such as MCM8 have been associated with KD susceptibility. Here we elaborate on the mechanistic pathway linking MCM8-mediated mitophagy dysfunction to persistent inflammation and vascular injury.

Mitophagy is a selective form of autophagy that eliminates damaged mitochondria; its dysfunction leads to mitochondrial DNA (mtDNA) release and activation of the cGAS-STING pathway [56]. Wei et al. (2024) found that serum mtDNA and 2′3′-cyclic GMP-AMP (2′3′-cGAMP) levels were significantlyelevated in acute KD patients; treatment with cyclosporine A markedly reduced mtDNA release and decreased cGAS-STING pathway protein expression [57].

Lin et al. (2023) reported that MCM8 mRNA expression in peripheral blood was significantly decreased in KD children with coronary artery aneurysms than in those without CALs, and type I interferon signaling genes were markedly upregulated; Mcm8-knockout mice showed substantially increased coronary arteritis area and p-IRF3 expression [36]. Marek-Iannucci et al. further confirmed that LCWE-injected mice exhibit impaired autophagy/mitophagy and elevated ROS; vascular smooth muscle cell-specific Atg16l1 deficiency and Parkin^−^/^−^ mice developed more severe lesions; and the mitochondrial-targeted antioxidant MitoQ attenuated vascular inflammation [19]. Mitophagy dysfunction leads to accumulation of damaged mitochondria and mtDNA release, activating the cGAS-STING pathway, which is a key event in the transition from inflammation to structural vascular injury.

#### 3.1.3. Autophagy–Inflammasome Crosstalk

There is a complex negative regulatory relationship between autophagy and inflammasomes. Latz et al. systematically described the inhibitory effect of autophagy on inflammasome activation [53]. Gupta et al. further delineated three mechanisms by which autophagy suppresses NLRP3 inflammasome activation: (1) clearance of damaged mitochondria, which reduces mtDNA release and ROS production; (2) direct degradation of NLRP3 inflammasome components; and (3) p62/SQSTM1-mediated selective autophagic clearance of ubiquitinated NLRP3 [54]. Conversely, inflammasome activation can also affect autophagy: IL-1β and other inflammatory cytokines induce cellular stress and modulate autophagic flux [58]. Recent reviews indicate that the functional status of the autophagy–inflammasome axis determines the outcome of inflammation—intact autophagy promotes resolution, whereas defective autophagy leads to persistent inflammation and tissue injury [59,60]. This “molecular switch” hypothesis provides a theoretical framework for understanding the transition from inflammation to vascular remodeling in IVIG-resistant patients.

Notably, a recent study by Atici et al. (2026) identified an impaired NAD^+^-SIRT1 axis in LCWE-induced KD vasculitis, demonstrating that SIRT1 activation promotes autophagy and mitophagy while reducing pro-inflammatory cytokine production [61]. This finding provides additional mechanistic insight into how metabolic regulation intersects with the autophagy–inflammasome axis in KD.

Beyond autophagy, neutrophil extracellular traps (NETs) represent another inflammatory effector that may contribute to vascular injury in KD.

### 3.2. Neutrophil Extracellular Trap (NET) Formation

#### 3.2.1. Discovery and Classical Mechanism of NETs

NETs are structures released by neutrophils, composed of a DNA scaffold and granule proteins (e.g., myeloperoxidase MPO, neutrophil elastase NE); they participate in pathogen clearance but can also mediate tissue injury [20]. NETs are DNA-protein structures released via peptidylarginine deiminase (PAD) 4-dependent chromatin decondensation (NETosis), which is a ROS-dependent programmed cell death distinct from apoptosis/necrosis that was first described for antimicrobial function [62,63,64].

#### 3.2.2. Controversy and Mechanistic Insights into NETs in KD Vasculitis

Jin et al. (2025) showed that serum NET markers (cfDNA, MPO, and NE) were significantly elevated in acute KD patients and correlated positively with CRP; children with CALs had higher NET levels compared with those without CALs [20]. However, correlation does not establish causation, and subsequent mechanistic studies have challenged the necessity of NETs in KD vasculitis. Domiciano et al. (2024) found that the pan-PAD inhibitor Cl-amidine significantly reduced coronary arteritis area in LCWE-induced KD mice; however, neutrophil-specific Padi4 knockout mice showed no difference in lesion area compared with wild-type mice, whereas the PAD2 inhibitor AFM30a also substantially reduced lesion area [65].

#### 3.2.3. Revision of Previous Understanding: NETs May Not Be Essential

PAD4-dependent NET formation may not be essential in KD vasculitis. The efficacy of PAD inhibitors in Padi4 knockout mice suggests functional redundancy between PAD2 and PAD4. Further mechanistic studies indicate that the protective effect of PAD inhibitors is likely mediated mainly through suppression of macrophage NLRP3 inflammasome activation rather than inhibition of NET formation [65]. This interpretation is consistent with the central pathogenic role of NLRP3 in KD [24] and suggests that PAD-mediated protein citrullination may indirectly influence inflammation by modulating macrophage function. Importantly, to date, no study has directly measured PAD2 or PAD4 expression or activity in coronary tissues from IVIG-resistant patients with active vasculitis. Until such data emerge, the therapeutic relevance of PAD inhibition in human KD remains speculative. Furthermore, whether the protective effects of PAD inhibitors in mouse models are mediated through NET inhibition, NLRP3 suppression, or both requires confirmation in human cells and tissues.

The above mechanisms contribute to persistent inflammation and endothelial activation. However, the transition to irreversible vascular remodeling requires a more fundamental change in endothelial cell identity: EndMT.

### 3.3. Endothelial-to-Mesenchymal Transition (EndMT) and Vascular Remodeling

#### 3.3.1. Classical Mechanism and Reversibility of EndMT

EndMT is the process by which endothelial cells lose their endothelial phenotype and acquire a mesenchymal phenotype (expressing α-SMA, vimentin) under inflammatory stimulation; it is a core event in vascular remodeling [20]. TGF-β signaling is a key driver of EndMT [66]. During EndMT, endothelial cells lose intercellular junctions and endothelial markers (VE-cadherin, CD31), gain mesenchymal markers (α-SMA, vimentin), and acquire migratory and invasive capabilities, a process involving activation of the transcription factors Snail, Slug, and Twist [67].

Early EndMT may be reversible upon removal of inflammatory stimuli, whereas late EndMT with fibrosis tends to become irreversible [66]. Clinically, CALs detected by echocardiography beyond 6–8 weeks after onset rarely regress completely, suggesting that the “point of no return” for EndMT may occur within the first month. This aligns with our three-stage model but requires direct temporal evidence. Based on available evidence, it is plausible that IVIG-resistant patients, who have higher and more prolonged levels of inflammatory cytokines (IL-1β, TNF, and IL-6) [31,68,69], may exhibit more pronounced EndMT.

#### 3.3.2. IL-1β/TNF Axis-Induced EndMT

Buthe et al. (2025) showed that treatment of HCAECs with a KD serum inflammatory matrix up-regulated ICAM-1 and VCAM-1 expression by 43.7-fold and 10.3-fold, respectively, while CD31 expression was decreased to 0.75-fold of untreated controls; IL-1R1 blockade substantially reduced ICAM-1 expression from 43.7-fold to 21.0-folds [21]. These findings establish that the IL-1β/TNF axis drives coronary endothelial activation and EndMT in a multimediator inflammatory environment.

#### 3.3.3. USP7-TGFβ2/SMAD Pathway

Deubiquitinating enzymes play critical roles in signal transduction. Qian et al. (2025) found that USP7 protein expression was significantly elevated in cardiac tissue from KD children and CAWS-induced KD mouse myocardium; USP7 knockout markedly reduced α-SMA-positive cells and collagen deposition area; the USP7 inhibitor P22077 significantly decreased EndMT markers [22]. This study revealed that USP7 promotes EndMT, cardiac fibrosis, and vascular remodeling by enhancing TGFβ2/SMAD2/3 signaling.

#### 3.3.4. Role of Myofibroblasts in Vascular Remodeling

Shimizu et al. (2013) reported that α-SMA-positive, smoothelin-negative myofibroblasts were present in the thickened intima of KD coronary artery aneurysms and co-expressed IL-17 and IL-6. TGF-β signaling has been implicated in the generation of myofibroblasts and may contribute to aneurysm formation [29]. Myofibroblast proliferation may contribute to structural vascular wall remodeling during the subacute/chronic phase. Together with the newly discovered USP7 mechanism, a relatively complete EndMT regulatory network emerges: inflammatory cytokines (IL-1β, TNF) activate endothelial cells and upregulate TGF-β signaling; USP7 stabilizes SMAD2/3 to amplify TGF-β signaling, driving EndMT; and myofibroblast proliferation leads to fibrosis and structural remodeling. 

### 3.4. Interactions Among the Three Mechanisms

Autophagy, NETs, and EndMT do not operate independently but form a complex interactive network that collectively determines the transition from inflammation to remodeling (Table 2). 

#### 3.4.1. Autophagy–NETs Crosstalk

Autophagy influences NET formation; the expression of autophagy–related genes (ATG5, ATG7) in neutrophils correlates with NET release. Conversely, NET components (e.g., elastase) can induce endothelial autophagy dysfunction, creating a vicious cycle [20,55]. Direct interventional studies on this crosstalk in KD models are lacking.

#### 3.4.2. Autophagy–EndMT Crosstalk

Autophagy exerts bidirectional regulation on EndMT. In early EndMT, moderate autophagy clears damaged organelles and delays the process; however, persistent autophagy dysfunction or excessive autophagy can promote EndMT. TGF-β signaling both induces EndMT and modulates autophagy, and the two pathways cross-regulate through SMAD and non-SMAD signaling. In KD-related studies, Qin et al. found that PBMC-induced endothelial cell autophagy enhancement is associated with endothelial dysfunction [55], however, the changes in autophagy levels during EndMT mediated by USP7 remain to be clarified. Based on the autophagy–inflammasome axis, it is hypothesized that autophagy dysfunction may indirectly upregulate TGF-β by increasing IL-1β production, thereby promoting EndMT.

#### 3.4.3. NETs-EndMT Crosstalk

NETs can activate endothelial cells and promote EndMT by releasing ROS, elastase, and pro-inflammatory cytokines. In KD, NET levels correlate with the degree of CALs [20,70]; it is speculated that NETs may participate in EndMT through activation of TGF-β signaling or induction of oxidative stress. Direct evidence for NET–EndMT crosstalk in KD is lacking and represents an important direction for future research.

Thus, the status of the autophagy–inflammasome axis may influence the propensity for NET formation and the threshold for EndMT initiation. Conversely, EndMT-derived extracellular matrix components and inflammatory mediators may feed back to regulate autophagy and NET release. 

## 4. Mechanistic Integration: Molecular Switches from Inflammation to Remodeling

### 4.1. The Autophagy–Inflammasome Axis as a Candidate Molecular Switch (Hypothesis-Driven Framework)

Based on the available evidence [53,54,58,59,60]—which is largely derived from non-KD models and correlative human studies—we propose that the “autophagy–inflammasome axis” could serve as a molecular switch determining whether inflammation persists or resolves. It is important to emphasize that this remains a hypothesis, not an established mechanism in IVIG-resistant KD. The core logic is as follows: when autophagy is intact, it clears damaged mitochondria, reduces mtDNA release, and directly degrades NLRP3 inflammasome components, thereby suppressing inflammasome activation and promoting resolution; when autophagy is defective, the accumulation of damaged mitochondria, increased ROS, and mtDNA release activate the NLRP3 inflammasome, leading to massive IL-1β production and amplification of inflammation. The key innovation of this hypothesis is elevating the functional status of autophagy from a “bystander” to a potential “active switch” that determines the outcome of inflammation, thereby providing a mechanistic explanation for why some IVIG-resistant patients progress to CALs while others do not. Testing this hypothesis will require direct measurement of autophagic flux and inflammasome activity in longitudinal cohorts of IVIG-resistant patients, as well as interventional studies in KD animal models with autophagy modulators.

The hierarchical relationships among the cGAS-STING axis, NLRP3 inflammasome activation, and NET-related pathways remain unresolved. Based on the available evidence, we propose the following hierarchical framework: (1) upstream—mitophagy dysfunction serves as the primary initiating event, leading to mtDNA release and cGAS-STING activation [38,57]; (2) midstream—cGAS-STING signaling amplifies type I interferon responses and primes NLRP3 inflammasome activation [38]; (3) downstream—NLRP3 inflammasome-derived IL-1β and IL-18 drive the bulk of the inflammatory response and endothelial dysfunction [24,51]; and (4) parallel (modulatory)—NETs may contribute to inflammation but appear to play a non-essential role, with PAD inhibitors’ protective effects likely mediated through macrophage NLRP3 suppression rather than NET inhibition per se [65]. This model positions mitophagy dysfunction as the primary driver, NLRP3 as the central effector, and NETs as a potentially redundant/modulatory pathway—a framework that now requires direct experimental validation in IVIG-resistant KD models.

In IVIG-resistant patients, autophagy dysfunction (particularly impaired mitophagy) may bias this switch toward “persistent inflammation”, subsequently driving vascular remodeling. The hypothesis generates three testable predictions: 

(1) IVIG-resistant patients with intact autophagy will have better resolution of inflammation and a lower incidence of CALs; 

(2) The severity of autophagy dysfunction will correlate with IL-1β levels and CALs risk in a dose-response manner; 

(3) In KD animal models, early induction of autophagy will reverse inflammasome activation and attenuate CALs, whereas autophagy inhibition will worsen lesions.

Indirect clinical evidence supports this hypothesis. A systematic review and meta-analysis by Liu et al. (2024) reported that lower albumin levels were significantly associated with increased risks of both IVIG resistance (OR = 0.83, 95% CI: 0.79–0.88) and coronary artery lesions (OR = 0.92, 95% CI: 0.87–0.96); elevated C-reactive protein-to-albumin ratio (CAR) was associated with increased risks of IVIG resistance (OR = 1.69, 95% CI: 1.39–2.05) and CALs (SMD = 0.52); and higher prognostic nutritional index (PNI) was associated with a decreased risk of CALs (OR = 0.82, 95% CI: 0.72–0.94) [71]. These markers may indirectly reflect autophagy status and systemic metabolic state, but their direct relationship with the autophagy–inflammasome axis requires further validation.

### 4.2. Inflammation-to-Remodeling Transition Model

**Figure 1:**
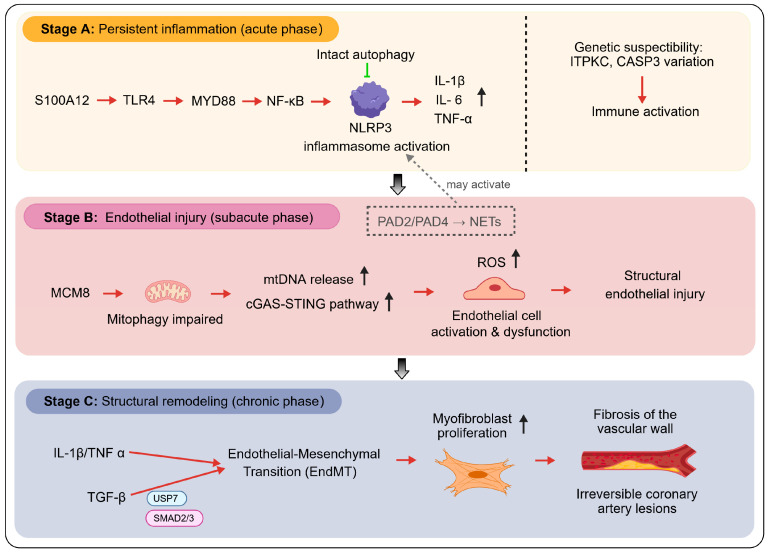
Three-stage transition model from inflammation to vascular remodeling in IVIG-resistant KD. The model proposes a sequential progression: Stage A: Persistent inflammation (acute phase, typically within the first week of fever), activation of the S100A12-TLR4-MYD88 axis, persistent NLRP3, and massive release of IL-1β, IL-6, and TNF-α. Genetic susceptibility factors (ITPKC, CASP3 variants) enhance immune activation. This stage is dominated by reversible inflammation and represents the optimal window for IVIG therapy. Stage B: Endothelial injury (subacute phase, corresponding to the period when IVIG resistance is identified) and autophagy dysfunction (MCM8-mediated impaired mitophagy) lead to mtDNA release and cGAS-STING activation, increased oxidative stress, and endothelial cell activation and dysfunction. Inflammation begins to transition toward structural endothelial injury. Stage C: Structural remodeling (chronic phase, with progressive vascular fibrosis) and IL-1β/TNF and TGF-β signals synergistically induce EndMT; USP7 stabilizes SMAD2/3 to amplify TGF-β signaling; myofibroblast proliferation leads to vascular wall fibrosis and irreversible CALs. Red arrows: promotion (e.g., inflammation, remodeling); green blunt-end lines: inhibition (e.g., autophagy suppresses NLRP3 inflammasome); and dashed box and dashed arrows: NETs role remains controversial (may act via NLRP3). Created with BioGDP.com [76]. The framework integrates evidence from animal models (LCWE/CAWS) and human cross-sectional studies; direct longitudinal evidence in IVIG-resistant patients is limited. Caution is warranted in extrapolating animal data to human IVIG resistance. The three stages represent a sequential progression; approximate timing is discussed in the main text.

**Figure 2:**
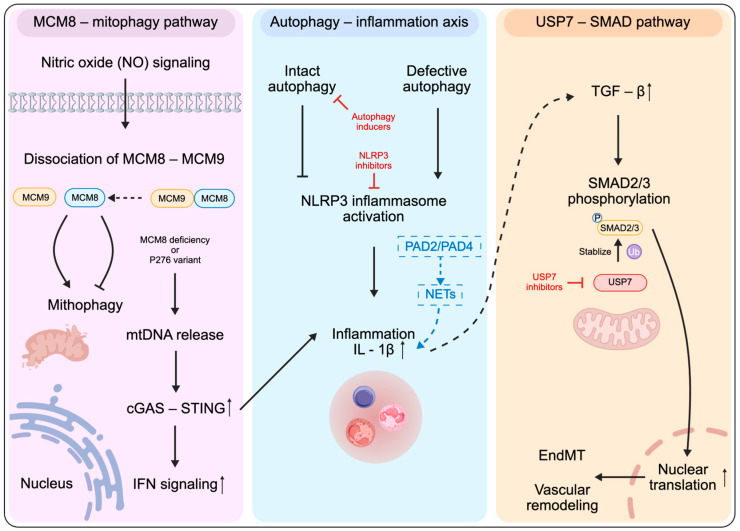
Molecular switch regulatory network: crosstalk among the autophagy–inflammasome axis, MCM8–mitophagy pathway, and USP7-SMAD pathway. This network illustrates the proposed crosstalk among three major pathways. (1) Autophagy–inflammasome axis (blue): intact autophagy inhibits IL-1β production; defective autophagy amplifies inflammation. (Note: PAD inhibitors may act via macrophage NLRP3 rather than NETs.) (2) MCM8–mitophagy pathway (purple): nitric oxide (NO) signaling promotes dissociation of the MCM8–MCM9 complex, allowing MCM8 to translocate to mitochondria and mediate mitophagy; MCM8 deficiency or the P276 variant impairs mitophagy, leading to mtDNA release and cGAS-STING activation, which promotes interferon signaling and inflammatory amplification. MCM8-mediated mitophagy pathway is primarily derived from mouse models; direct evidence in human IVIG-resistant KD is limited. (3) USP7-SMAD pathway (orange): inflammatory cytokines promote TGF-β production, which activates SMAD2/3 phosphorylation; USP7 deubiquitinates and stabilizes SMAD2/3, enhancing nuclear translocation and driving EndMT and vascular remodeling. Solid arrows indicate positive regulation; dashed arrows indicate indirect regulation; and blunt-ended lines indicate inhibition. Potential intervention targets are marked in red: USP7 inhibitors, NLRP3 inhibitors, and autophagy inducers. ↑ indicates up-regulation. Most pathways are supported by animal model and in vitro data; human validation is required. Created with BioGDP.com [76].

Integrating the above mechanisms, we propose a three-stage model of coronary transition from inflammation to remodeling in an IVIG-resistant KD (Figure 1) and a molecular switch network (Figure 2).

The three-stage model depicts a temporal sequence from acute inflammation to chronic vascular remodeling. In clinical practice, IVIG is recommended within the first 10 days of fever, ideally between days 5 and 7 [2,6]. IVIG resistance is defined as persistent or recrudescent fever ≥36 h after completion of initial IVIG infusion [2,72,73]. For patients treated on day 7 of fever, this resistance identification window corresponds approximately to fever days 8.5–9.5. Regarding the timeline of CALs dynamics, coronary artery involvement is usually maximal within 6–8 weeks after the acute episode [9]; some lesions begin to regress as early as 2 weeks from onset, and 63.4% of lesions have regressed by 8 weeks [74]. Complete regression rates increase over time: 39.2% within 4 weeks, 59.2% within 8 weeks, and 70.0% within 16 weeks [75].

### 4.3. Candidate Molecular Switches

Table 3 lists candidate molecular switches in the inflammation-to-remodeling transition.

### 4.4. Clinical Outlook: Potential Biomarkers and Therapeutic Targets

Based on the mechanistic framework, the following have the highest translational potential.

#### 4.4.1. First-Priority Biomarkers (Stronger Evidence)

S100A12 has emerged as a robust biomarker for IVIG resistance. Wu et al. (2024) demonstrated that S100A12 mRNA expression in PBMCs was significantly elevated in IVIG-resistant patients, and a relative S100A12 expression ≥10.224 was an independent risk factor for IVIG resistance (OR =1.079, 95% CI: 1.035–1.124) [52]. Mechanistically, S100A12 activates the TLR4-MYD88-NF-κB axis, driving a sustained inflammatory response that may override IVIG’s anti-inflammatory effects [31].

IL-1β and IL-18, as direct products of NLRP3 inflammasome activation, are consistently elevated in IVIG-resistant KD patients [48,50]. Serum IL-1β levels correlate positively with CRP and CALs severity [50]. These cytokines are not only biomarkers but also direct mediators of vascular injury.

MCM8 expression is reduced in KD patients with CALs, and the MCM8-P276 variant (enriched in East Asian populations) is associated with reduced mitophagy capacity and KD susceptibility [38]. While direct evidence linking MCM8 to IVIG resistance is currently limited, its role in sustaining inflammation through impaired mitophagy makes it a promising candidate biomarker for identifying patients at risk of poor outcomes.

Genetic variants including ITPKC (rs28493229), CASP3 (rs113420705), and FCGR2A (rs1801274) have been directly associated with IVIG resistance and CALs risk [26,27,28]. However, it should be noted that a large 2024 multi-cohort study found that FCGR2/3 polymorphisms, while associated with KD susceptibility, did not significantly predict IVIG resistance or CALs risk in meta-analyses [10], highlighting the need for population-specific validation.

Nutritional-inflammatory markers including hypoalbuminemia, elevated CAR, and low PNI are accessible, cost-effective predictors of IVIG resistance and CALs [71]. These markers may indirectly reflect systemic metabolic stress and autophagy status.

#### 4.4.2. Second-Priority Biomarkers (Preliminary)

LC3-II and p62 are conventional autophagy markers. However, their utility as biomarkers in KD is complicated by the divergent directions of autophagic change reported across different cell types and disease stages [19,55]. Longitudinal studies measuring autophagic flux in PBMCs and endothelial cells are needed.

USP7 is elevated in KD cardiac tissue and promotes EndMT through SMAD2/3 stabilization [22]. While its association with IVIG resistance has not been directly studied, its role in driving vascular remodeling makes it a candidate biomarker for CALs progression.

Soluble CD31 and VE-cadherin are shed from endothelial cells during EndMT and may reflect the extent of endothelial-to-mesenchymal transition [21].

#### 4.4.3. Therapeutic Targets

Autophagy inducers have shown preclinical efficacy: In LCWE-induced KD mice, metformin substantially reduced coronary arteritis scores, whereas the mitochondrial-targeted antioxidant MitoQ attenuated vascular inflammation and ROS production [19]. A recent double-blind randomized trial of MitoQ in dilated cardiomyopathy patients (Halliday et al., 2026) demonstrated its safety and feasibility in cardiovascular disease [77], supporting its potential repurposing for KD. However, no studies have specifically investigated metformin or MitoQ in IVIG-resistant KD, and their efficacy in the context of IVIG resistance requires dedicated investigation.

NLRP3 inhibitors: MCC950 has shown anti-inflammatory effects in preclinical models but was discontinued due to hepatotoxicity [78]. CY-09 and OLT1177 are under investigation [79]. The ROS-TXNIP-NLRP3 inflammasome axis has recently been implicated in endothelial dysfunction in KD [80], providing additional rationale for NLRP3 targeting.

USP7 inhibitors: P22077, FT671, and XL177A have demonstrated anti-EndMT and anti-inflammatory effects in KD model mice [19,81,82]. USP7 inhibition represents a novel strategy for preventing vascular remodeling by blocking SMAD2/3 stabilization and downstream EndMT.

IL-1 receptor antagonist anakinra: Anakinra directly blocks IL-1 signaling downstream of NLRP3 inflammasome activation [83]. The ongoing ANAKID trial is expected to provide higher-level evidence. Anakinra is currently not FDA-approved for KD but is used off-label in refractory cases. 

#### 4.4.4. IL-1 Blockade as a Mechanism-Validating Therapy

Given the central role of NLRP3 inflammasome-derived IL-1β in driving KD vasculitis [24], the IL-1 receptor antagonist anakinra has emerged as a promising salvage therapy for IVIG-resistant KD. Recent case series and multicenter reports have shown that anakinra improves inflammatory markers and halts coronary artery aneurysm progression in patients failing IVIG and corticosteroids [84,85]. A multicenter Italian case series including eight infants (≤6 months) with IVIG-resistant KD and CALs reported that anakinra (mainly intravenous, median dose 8.5 mg/kg/day) led to complete CAL resolution in five patients and improvement in two, with only one treatment-related adverse event [84]. From a mechanistic perspective, IL-1 blockade acts downstream of the proposed autophagy–inflammasome axis. Regardless of whether the “molecular switch” is stuck in the “on” position due to autophagy dysfunction, anakinra directly intercepts the IL-1β signal. If anakinra demonstrates greater efficacy in patients with biomarkers of autophagy dysfunction (e.g., elevated mtDNA, reduced MCM8 expression), this would provide indirect validation of the axis hypothesis. 

## 5. Limitations and Future Directions

### 5.1. Limitations of Current Research

#### 5.1.1. Discrepancies Between Animal Models and Human Disease

KD mechanistic studies rely mainly on LCWE- or CAWS-induced mouse models. While these models recapitulate certain pathological features (e.g., coronary arteritis), they have notable limitations: (1) the LCWE model is insensitive to IVIG, failing to fully reproduce the IVIG response seen in humans; (2) mouse models lack typical clinical manifestations of KD (e.g., mucositis, rash); and (3) the coronary artery anatomy differs between mice and humans. Importantly, most mechanistic studies cited in this review (e.g., PAD inhibitor experiments, autophagy manipulation in LCWE mice) were conducted without IVIG co-administration, limiting their translational relevance to the specific context of IVIG-resistant patients. Future animal models that incorporate IVIG treatment and selectively examine non-responders are urgently needed.

#### 5.1.2. Scarcity of Clinical Samples from IVIG-Resistant Patients

IVIG resistance occurs in only approximately 14% of KD children [9], and the incidence of coronary artery aneurysms is substantially higher in this subgroup (25–35% vs. 3–5%) [2], making sample collection difficult. Most existing studies are based on small cohorts (average <100 patients) and lack large-scale, multicenter prospective designs, hindering direct validation of mechanistic findings in human samples.

#### 5.1.3. Gap Between Single-Mechanism and Integrated Multi-Mechanism Studies

Current research has largely focused on individual pathways; longitudinal studies examining the temporal sequence and causal relationships among autophagy, NETs, and EndMT are lacking. Despite the complex crosstalk among these three pathways, integrated studies in KD are virtually absent.

#### 5.1.4. Heterogeneity of CALs Phenotypes

Whether the mechanisms proposed herein (particularly mitophagy dysfunction and EndMT) differ between transient coronary dilation and giant aneurysms remains unknown, as longitudinal mechanistic stratification of CALs phenotypes has not been performed. Additionally, host genetic factors likely contribute to the heterogeneous treatment responses observed in KD, with ITPKC, CASP3, FCGR2A, and TSPAN5 variants directly linked to IVIG resistance [26,27,28,40], whereas MCM8 has been associated with KD susceptibility without an established relationship to IVIG resistance [38]. Critically, whether these genetic variants modulate the threshold of the proposed “autophagy–inflammasome molecular switch”—for example, by influencing mitophagy efficiency or NLRP3 activation propensity—has not been investigated. This knowledge gap precludes accurate genetic stratification of patients at risk for progression to irreversible CALs, and represents a priority area for future mechanistic and translational research.

#### 5.1.5. Need for Direct Validation of the Autophagy–Inflammasome Axis Hypothesis

The “autophagy–inflammasome axis” as a molecular switch is a central hypothesis of this review. However, direct evidence for this switch in IVIG-resistant KD is currently lacking. Most supporting data come from studies in non-KD inflammatory models (e.g., LPS-stimulated macrophages) or from cross-sectional observations in KD patients that show correlations rather than causal relationships. Specifically, no study has yet simultaneously measured autophagic activity (e.g., LC3-II flux, p62 turnover) and NLRP3 inflammasome activation (e.g., ASC speck formation, caspase-1 activity, and IL-1β secretion) in the same cohort of IVIG-resistant patients over time. Furthermore, it remains unknown whether the severity of autophagy dysfunction quantitatively correlates with IL-1β levels and CALs risk in a dose–response manner, which is a key prediction of the hypothesis. Future prospective studies should incorporate dynamic monitoring of autophagy markers (e.g., plasma LC3-II, beclin-1, and MCM8 expression) alongside inflammasome products (e.g., IL-1β, IL-18, and ASC) at multiple time points (pre-IVIG, post-IVIG, subacute, and convalescent) to directly test this hypothesis. Animal experiments using conditional knockout of autophagy genes (e.g., Atg16l1 in endothelial cells or myeloid cells) combined with IVIG administration would also help establish causality.

### 5.2. Future Directions

#### 5.2.1. Deepening Mechanistic Studies

(1) Autophagy–inflammasome crosstalk: Can autophagic activity serve as a biomarker to distinguish “reversible inflammation” from “irreversible injury”? Prospective cohort studies with dynamic monitoring of autophagy markers and inflammasome products are needed.

(2) True contribution of NETs in human KD: Given that PAD4-dependent NET formation is not essential in mouse models, direct validation in human IVIG-resistant patients is necessary, including analysis of PAD2/PAD4 expression and their relationship with NLRP3.

(3) Upstream integrating signals for EndMT: How do IL-1β/TNF and TGF-β signals synergistically induce EndMT? Multi-factor stimulation of endothelial cell models is needed to systematically study crosstalk among signaling pathways.

#### 5.2.2. Multicenter Prospective Cohort Studies and Integration of Emerging Technologies

Multicenter prospective cohort studies should validate the autophagy–inflammasome axis hypothesis. Peripheral blood should be collected at key time points (admission, post-IVIG, subacute phase, and convalescent phase) for assessment of autophagic function, inflammasome activation, EndMT markers, and genetic profiling. Mixed-effects models and structural equation modeling should be used to analyze causal relationships. In addition, single-cell multi-omics analysis of extreme-phenotype patients and the use of human coronary artery organoids or vessel-on-a-chip models to simulate the inflammation-to-remodeling transition will provide high-throughput platforms for mechanistic studies and drug screening.

## 6. Conclusions

This review focuses on three mechanistic pathways: (1) autophagy dysfunction (particularly MCM8-mediated impaired mitophagy) that mediates persistent inflammation via activation of the cGAS-STING pathway; (2) the controversial role of NET formation in KD vasculitis, with PAD2/PAD4 possibly playing redundant roles through the NLRP3 inflammasome; and (3) EndMT as a core event in vascular remodeling, regulated by the IL-1β/TNF axis and the USP7-TGFβ2/SMAD pathway.

The core contributions of this review are: hypothesizing the “autophagy–inflammasome axis” as a candidate molecular switch that may govern inflammation persistence versus resolution, while explicitly acknowledging that direct validation is required and constructing a three-stage transition model from inflammatory cascade to vascular remodeling. This framework suggests that early identification of markers of autophagy dysfunction (e.g., decreased MCM8 expression, elevated mtDNA) may help screen high-risk patients for IVIG resistance and provides a theoretical basis for intervention strategies targeting NLRP3 or USP7. Future validation of these hypotheses will require multicenter prospective cohort studies integrated with single-cell multi-omics and organoid technologies.

## Figures and Tables

**Table 1:** Genetic susceptibility loci for Kawasaki disease and their association with IVIG resistance.

Pathway	Gene/Locus	Functional Significance	Association with KD Susceptibility	Direct Association with IVIG Resistance
T-cell related	ITPKC (rs28493229)	Regulates calcium signaling and T-cell activation	OR = 1.64	Yes [26]
	CASP3 (rs113420705)	Regulates apoptosis and inflammatory clearance	OR = 1.41	Yes (1.6-fold increased risk) [27]
	ORAI1 (rs3741596)	T-cell calcium signaling and activation	Associated with KD	To be validated * [34]
	TNF (rs1800629)	Pro-inflammatory cytokine	Associated with KD	To be validated * [34]
B-cell related	FCGR2A (rs1801274)	Modulates IgG receptor affinity	OR = 1.31	Yes (1.5-fold increased risk) [28]
	CD40 (rs153045)	B-cell activation and immune response	OR = 1.23	Indirect (via CALs risk) [35]
	BLK (rs2736340)	B-cell signaling	Associated with KD	To be validated * [34]
Mitochondrial/other	MCM8 (P276 variant)	Mediates mitophagy	Associated with KD	To be studied † [36]
	NDUFA5	Mitochondrial complex I subunit	Associated with CALs	To be studied † [37]
	TSPAN5	Tetraspanin, cell signaling	Associated with KD and IVIG resistance	Yes [38]
	VEGFA	Vascular endothelial growth factor	Associated with CALs	To be studied † [39]
	20q13 region	Unknown function	Associated with CALs	To be studied † [40]

Note: * To be validated: preliminary association, needs replication. † To be studied: reported in KD, no direct IVIG resistance evidence yet. For a detailed discussion of MCM8 and mitophagy, refer to Section 3.1, “Autophagy Dysfunction and Impaired Mitophagy”.

**Table 2:** Key findings on autophagy, NETs and EndMT in Kawasaki disease.

Mechanism	Key Findings	Association with IVIG Resistance/CALs	Representative Reference
Autophagy	KD PBMCs induce HCAEC autophagy and increase chemokine/cytokine secretion; 3-MA partially reverses	Autophagy dysfunction linked to resistance and endothelial injury	Qin et al., Transl Pediatr, 2021 [55]
	MCM8-mediated mitophagy dysfunction activates cGAS-STING; MCM8-P276 variant increases susceptibility	Impaired mitophagy drives persistent inflammation, associated with CALs	Lin et al., Nat Cardiovasc Res, 2023 [36]
	LCWE mice show impaired autophagy/mitophagy; ATG16L1 or Parkin deficiency worsens lesions	Autophagy induction (metformin, MitoQ) attenuates cardiovascular inflammation	Marek-Iannucci et al., JCI Insight, 2021 [19]
	Elevated serum mtDNA and 2′3′-cGAMP in KD; cyclosporine A blocks pathways	Mitophagy dysfunction causes mtDNA leakage, exacerbates inflammation	Wei et al., Cell Commun Signal, 2024 [57]
	Autophagy clears damaged mitochondria and degrades NLRP3; dysfunction leads to sustained IL-1β	Autophagy–inflammasome axis as molecular switch	Gupta et al., Immunol Rev, 2025 [54]
NETs	Pan-PAD inhibitor reduces lesions; Padi4 knockout is ineffective; PAD2 inhibitor is effective	NETs not essential; PAD2/PAD4 redundant	Domiciano et al., Clin Exp Immunol, 2024 [65]
	Increased NET formation in acute KD, correlates with vascular injury	NETs may participate in injury; the mechanism needs validation	Jin et al., Int Immunopharmacol, 2025 [20]
EndMT	KD serum inflammatory matrix induces EndMT in HCAECs; IL-1R1 blockade is the most effective	IL-1β/TNF axis drives EndMT, which is key for inflammation-to-remodeling transition	Buthe et al., ACR Open Rheumatol, 2025 [21]
	USP7 elevated in KD; USP7 stabilizes SMAD2/3 to enhance TGF-β signaling; inhibitor reduces EndMT	USP7 is a key regulator of EndMT and promotes vascular remodeling	Qian et al., Int Immunopharmacol, 2025 [22]
	α-SMA^+^ myofibroblasts are found in KD aneurysmal intima; TGF-β signaling is implicated in their generation	Myofibroblast generation is central to arterial remodeling in KD.	Shimizu et al., Hum Pathol, 2013 [29]

Note: IVIG: intravenous immunoglobulin; CALs: coronary artery lesions; KD: Kawasaki disease; PBMCs: peripheral blood mononuclear cells; HCAECs: human coronary artery endothelial cells; LCWE: lactobacillus cell wall extract; NETs: neutrophil extracellular traps; EndMT: endothelial–mesenchymal transition; PAD: peptidylarginine deiminase; USP7: ubiquitin-specific protease 7; SMAD: SMAD and MAD-related protein; and α-SMA: alpha-smooth muscle actin.

**Table 3:** Summary of molecular markers associated with IVIG resistance and CALs.

Category	Molecule/Marker	Mechanism	Association with IVIG Resistance	Association with CALs	Sample	Representative Reference
Inflammation	S100A12	Activates TLR4-MYD88, drives inflammatory storm	Enhanced in non-responders	Elevated	Serum/PBMCs	Feng et al., 2025 [31]
	IL-1β, IL-18	NLRP3 inflammasome products	Pyroptosis activation linked to resistance	Promotes endothelial injury	Serum	Wang et al., 2025 [50]
Autophagy	LC3-II, p62	Autophagy markers	PBMCs from resistant patients induce HCAEC autophagy abnormality	Dysfunction linked to CALs	Cells/tissue	Qin et al., 2021 [55]
	MCM8	Mediates mitophagy	Indirect evidence	Reduced in CAL patients	Blood/tissue	Lin et al., 2023 [36]
EndMT	USP7	Deubiquitinase, stabilizes SMAD2/3	To be studied ^†^	Elevated in KD, promotes remodeling	Heart tissue	Qian et al., 2025 [22]
	Soluble CD31, VE-cadherin	Endothelial markers, downregulated during EndMT	To be studied ^†^	Reflects endothelial dysfunction and EndMT	Serum/plasma	Buthe et al., 2025 [21]
Genetic	ITPKC (rs28493229)	Regulates T-cell calcium signaling	Significantly associated	Associated with CALs risk	Blood DNA	Onouchi et al., 2008 [26]
	CASP3 (rs113420705)	Regulates apoptosis	1.6-fold increased resistance risk	Associated with severity	Blood DNA	Onouchi et al., 2010 [27]
	FCGR2A (rs1801274)	Modulates IgG receptor affinity	1.5-fold increased resistance risk	Predicts CALs progression	Blood DNA	Khor et al., 2011 [28]
Nutritional	Albumin (ALB)	Reflects nutritional/inflammatory status	Hypoalbuminemia predicts resistance	Hypoalbuminemia linked to CALs	Serum	Liu et al., 2024 [71]
	C-reactive protein/albumin ratio (CAR)	Integrates inflammation and nutrition	Elevated CAR linked to resistance	Elevated CAR linked to CALs	Serum	Liu et al., 2024 [71]
	Prognostic nutritional index (PNI)	Combines albumin and lymphocyte count	Low PNI linked to resistance	Low PNI linked to CALs	Serum	Liu et al., 2024 [71]

Notes: ^†^ To be studied: reported in KD, no direct IVIG resistance evidence yet.

## Data Availability

No data was used for the research described in the article.
